# Innovative Applications of *Tenebrio molitor* Larvae in Food Product Development: A Comprehensive Review

**DOI:** 10.3390/foods12234223

**Published:** 2023-11-22

**Authors:** Konstantina Kotsou, Theodoros Chatzimitakos, Vassilis Athanasiadis, Eleni Bozinou, Christos G. Athanassiou, Stavros I. Lalas

**Affiliations:** 1 Department of Food Science and Nutrition, University of Thessaly, Terma N. Temponera Str., 43100 Karditsa, Greece; kkotsou@agr.uth.gr (K.K.); tchatzimitakos@uth.gr (T.C.); vaathanasiadis@uth.gr (V.A.); empozinou@uth.gr (E.B.); 2 Laboratory of Entomology and Agricultural Zoology, Department of Agriculture, Crop Production and Rural Environment, School of Agricultural Sciences, University of Thessaly, Phytokou Str., 38446 Volos, Greece; athanassiou@uth.gr

**Keywords:** Tenebrionidae, edible insects, food additive, food products, nutritional enrichment

## Abstract

The utilization of alternative and sustainable food sources has garnered significant interest as a means to address the challenges of food security and environmental sustainability. *Tenebrio molitor* larvae, commonly known as mealworms, have emerged as a promising candidate in this context, as they are a rich source of nutrients and can be reared with relatively low resource input. This review article presents an in-depth analysis of the diverse range of food products developed using *T. molitor* larvae and the distinctive properties they bestow on these products. The review encompasses an exploration of the nutritional composition of the larvae, emphasizing their rich protein content, balanced amino acid profile, fatty acids with health benefits, vitamins, and minerals. It delves into how these attributes have been harnessed to enhance the nutritional value of a variety of food items, ranging from protein-rich snacks and energy bars to pasta, bakery goods, etc. Each of these applications is discussed with regard to how *T. molitor* larvae contribute to the nutritional content and sensory characteristics of the final product. Furthermore, this review sheds light on the innovative techniques and processing methods employed to incorporate *T. molitor* larvae into different food matrices. It addresses challenges related to taste, texture, and appearance that have been encountered and the strategies devised to overcome related problems. Overall, this comprehensive review elucidates the diverse food products that have been developed utilizing *T. molitor* larvae as a key ingredient. Highlighting the nutritional, sensory, and sustainability aspects of these products, this review offers valuable insights to harness the potential of this alternative protein source to meet the evolving needs of modern food systems.

## 1. Introduction

The exploration of alternative food sources for human consumption has garnered significant attention due to several compelling factors, with most of them revolving around overpopulation and the high nutritional value of these novel food sources. This ongoing issue of overpopulation, which has been a topic of concern in the research community since 1997 [1], naturally correlates with an increasing demand for conventional protein sources, namely, meat and fish [2,3,4,5]. The expansion of livestock farming, driven by this escalating demand, poses the risk of encroaching on previously uncultivated land, exacerbating the issue of land use, considering that 75% of arable land is currently dedicated to livestock activities [6,7]. Moreover, the livestock sector is a major contributor, accounting for 14.5% of total anthropogenic greenhouse gas emissions [8], raising concerns about the environmental impact of this sector, which is likely to escalate with an increase in livestock units [9]. In the context of fish as a key source of protein, numerous fish species are endangered worldwide as a consequence of overfishing, pollution, coastal development, climate change and other anthropogenic actions [10]. An example is *Chondrichthyes*. The *Chondrichthyes* species is in danger, since 32.6% (391 out of 1199 species) are threatened with complete extinction due to overfishing [11]. This overexploitation of marine resources has resulted in the degradation of several marine habitats [11,12,13]. Given these ecological and sustainability concerns surrounding conventional protein sources, the scientific community has shifted its focus toward identifying alternative, sustainable sources of protein. Among these, insects have emerged as one of the most promising options [14,15,16,17,18].

Insects exhibit distinct advantages from economic and ecological standpoints. They require considerably less space, minimal water (relying primarily on moisture), and reduced feed to complete their biological life cycle compared to traditional protein sources [19,20,21,22]. However, it is noteworthy that the concept of insects as a food source is not entirely novel. Recent archaeological evidence from Tanzania suggests that insects played a crucial role in the nutrition of early humans, dating back 1.8 million years [23]. Additionally, historical depictions, such as those found in the Artamila caves in northern Spain (3000–9000 BC), portray the collection of bee nests and larvae, further highlighting the historical significance of insect consumption [24]. Despite this historical practice, insect consumption has declined significantly over time and is now primarily narrowed to traditional practices in certain regions like Southeast Asia and Australia [25,26]. In Europe, insect consumption remains relatively rare, largely due to “neophobia” surrounding insect consumption in any form [27,28,29,30]. Nevertheless, as of 2021, three insect species have received approval for safe human consumption by the European Parliament and Council [31]. In particular, on 12 November 2021 they authorized the placing on the market of frozen, dried, and powdered forms of *Locusta migratoria* Linnaeus (Orthoptera: Acrididae) [32], on 1 June 2021 the placing on the market of dried *Tenebrio molitor* Linnaeus (Coleoptera: Tenebrionidae) larvae as a novel food [33] and, most recently, on 5 January 2023, the frozen, paste, dried and powder forms of *Alphitobius diaperinus* Panzer (Coleoptera: Tenebrionidae) larvae (lesser mealworm) [34].

One of the approved species, *Tenebrio molitor* Linnaeus (Coleoptera: Tenebrionidae), is particularly noteworthy for its potential to be more readily accepted by consumers, especially in Europe [35,36,37,38]. For instance, Europeans tend to find the larvae of *T. molitor* more appealing than alternatives like cockroaches or ants [39]. Moreover, the rearing of *T. molitor* shows great promise in the rapidly expanding, global, edible insect market, where it is already being widely produced as feed for fish and poultry [40,41,42,43].

A comparative analysis of the rearing of *T. molitor* and *Alphitobius diaperinus* Panzer (Coleoptera: Tenebrionidae) reveals several advantages of the former. *T. molitor* larvae complete their development and growth in a shorter time frame, boasting a higher survival rate and greater weight compared to *A. diaperinus* [44]. Specifically, the survival rate of *T. molitor* larvae is as high as 98.8%, while that of *A. diaperinus* ranges from 88 to 95%. Additionally, the weight of *T. molitor* larvae is substantially greater, compared to the weight of *A. diaperinus* larvae [45,46,47]. This disparity is expected, given that *T. molitor* is larger than *A. diaperinus* at all stages of development [48,49]. When comparing the rearing of *T. molitor* to that of *Locusta migratoria* Linnaeus (Orthoptera: Acrididae), it becomes evident that *T. molitor* rearing is both faster and more straightforward. The development of *T. molitor* larvae is completed in just 2–3 months, whereas *L. migratoria* larvae require 2–4 months for their development [50,51,52]. This efficiency in rearing further highlights the potential of *T. molitor* as a viable and sustainable protein source for human consumption, particularly in regions where insect consumption is gaining recognition as an environmentally responsible dietary choice.

The pursuit of alternative food sources, driven by concerns for overpopulation and the environmental impact of traditional protein sources, has led to the exploration of insects as a sustainable protein option. *T. molitor*, in particular, holds promise due to its appeal to European consumers, efficient rearing, and favorable growth characteristics compared to other insect species. This comprehensive review aims to provide a thorough exploration of the diverse applications of *T. molitor* larvae, in the realm of food product development. It endeavors to elucidate the multifaceted potential of *T. molitor* larvae as a sustainable and nutritious protein source for human consumption. By comprehensively reviewing the existing research and applications of *T. molitor* larvae in food product development, this review aims to provide valuable insights for researchers, food industry professionals, and policymakers. Furthermore, it underscores the need for continued exploration and development in this field to advance the sustainable food options required to meet the demands of a growing global population while mitigating environmental concerns.

## 2. Nutritional Value of *T. molitor*

As is frequently mentioned, their high nutritional value is the reason that led researchers to extensively study the larvae of *T. molitor*. This species is, to this day, the main subject of research since it is characterized by its content of essential nutrients, including crude protein, essential amino acids (EAAs), fat, and essential fatty acids [24,53,54,55,56,57]. The quantification of the crude protein of *T. molitor* and its flour (dried and finely ground larvae) has received special attention. Due to this, several analytical methods have been used, such as the combustion (Dumas) method [56], Randall method [35], Kjeldahl method [58], elemental analysis method [59], Bradford method [60], and precipitation [61], yielding a variety of results, ranging from 36.8–75.1%. This variation can be attributed to the different rearing conditions of each *T. molitor* larvae. At this point, it is worth emphasizing that conventional animal, protein-rich foods, such as beef, chicken, and tuna, contain 21.4, 19.4 [62], and 22.7% crude protein [63], respectively, values that can easily be considered low compared to the protein content of *T. molitor* larvae. In Table 1 the crude protein values contained in *T. molitor* larvae according to various surveys are presented.

Histidine (His), isoleucine (Iso), leucine (Leu), lysine (Lys), methionine (Met), phenylalanine (Phe), threonine (Thr), tryptophan (Trp), and valine (Val) are nine amino acids that cannot be synthesized by mammals and they must be obtained through food consumption. Therefore, they are called dietarily essential, indispensable nutrients or essential amino acids (EAAs) [64]. *T. molitor* larvae contain large amounts of Leu, Val, and Lys but low amounts of His, Met, and Trp EAAs, with Trp rarely occurring in these insects [55]. Similarly, to crude protein, EAA concentrations are influenced by the rearing conditions of the *T. molitor* larvae, temperature, humidity, feed substrate and rearing region [65,66]. For instance, Lys quantities in *T. molitor* larvae can range from 1.6 to 5.8%, Thr quantities can range from 1.3 to 4.3%, Met quantities can range from 0.6 to 2.2% and Trp quantities can range from 0.02 to 1.9% [35,42,43,44,45]. More details for the quantities of the EAAs are presented in Table 2.

The second most plentiful nutrient in the composition of *T. molitor* larvae is crude fat [70], which exhibits variability in values depending on the content of other nutrients and the processing method [55]. Predominantly, these larvae contain about 27% fat, whereas the percentage was found to range from 19.1 to 32.2% [56,57,58,59,60]. In Table 1, a detailed presentation of the crude fat values, with the corresponding crude protein content, is shown. In addition to EAAs, in *T. molitor* larvae, essential fatty acids (EFAs), such as linoleic acid (ω-6 group) and alpha-linolenic acid (ω-3 group) [71], are also detected [72]. Humans cannot produce these EFAs and thus must be obtained through the diet they are based on. The fatty acid composition of *T. molitor* larvae is presented in Table 3. As far as linolenic acid (C18:2 ω-6) is concerned, it is present in high amounts in *T. molitor* larvae, ranging from 11.5 to 48.1%. In contrast, as far as the content of alpha-linolenic acid (C18:3 ω-3) in larvae is concerned, it covers a range of values, from 0.2 to 2.3%. Furthermore, considerable quantities of fatty acids such as myristic acid (C14:0), palmitic acid (C16:0), palmitoleic acid (C16:1), and ω-9 oleic acid (C18:1) are also detected [38,42,45,46,47].

Crude ash constitutes one more important aspect of *T. molitor* larvae, which is tested in all studies investigating the nutritional value of *T. molitor* larvae. Crude ash refers to the total of all mineral elements such as phosphorus (P), calcium (Ca), sodium (Na), and magnesium (Mg) [73]. The crude ash as presented in Table 1 ranges from about 1.0 to 4.2% [56,57,58,59,60] and is also affected by the composition of the other nutrients, crude protein, and crude fat. Regarding, the minerals contained in *T. molitor* larvae were identified and quantified in a number of studies. In particular, the P content of larvae has recorded values up to about 1.0% [74], Ca content up to 0.5% [58], Na content up to 0.4% [57], Mg content up to 1.6% [58] and potassium (K) content up to about 1.0% [57]. In addition, *T. molitor* larvae contain up to 100.02 mg/kg of iron (Fe), up to 117.4 mg/kg, of zinc (Zn), and 20.0 mg/kg of copper (Cu) [74].

## 3. *Tenebrio molitor* and Its Derivatives as Innovative Products for Human Consumption

As stated in the introduction, humans have been consuming insects in their daily diet since ancient times [23]. However, it is presumed that, at that time, insects were mainly eaten raw. In the modern world, the habit of eating insects, known as entomophagy, was first described in 1975 by Meyer-Rochow, in which edible insects were proposed as a future dietary solution [75]. Nowadays, in Western countries the predominant consumption way of *T. molitor* larvae is in the form of ‘flour’ [76]. The preparation method of this flour has been relatively similar in all investigations to date. First, the larvae are dried either using lyophilization (e.g., lyophilizer for 48 h at a temperature of −96 °C and vacuum) [77] or an oven (e.g., air circulation oven at 40 °C for 48 h) [78] and then pulverized until they are in powder form. Consequently, most of the products obtained from mealworm larvae are bakery products, ranging from bread to pastries [79]. 

Moreover, *T. molitor* larvae are also well known for their high oil and fatty acid content [55]. Various methods and solvents carry out the extraction of the oil. For instance, the use of petroleum ether as solvent and extraction using a Soxhlet device at 50 °C for 6 h and evaporation with a rotary evaporator has been proposed [80]. Another method is the use of supercritical carbon dioxide (CO_2_) extraction, where for maximum degreasing (95%) pressure at 400/250 bar, a temperature of 45 °C and an extraction time of 105 min was applied [81]. In addition, extraction with hydroethanolic solvent (20:80, *v*/*v*) under continuous stirring (150 rpm) for 3 days has been proposed [78]. Alternatively, extraction using stirring at 40 °C and evaporation of the solvent with a rotary evaporator has also been proposed [60].

### 3.1. Bakery Goods

#### 3.1.1. Bread

Kim et al. [82] pioneered the incorporation of flour from *T. molitor* larvae for bread production, marking a significant milestone in the exploration of alternative protein-rich ingredients for bakery products. Their study involved the creation of three separate bread formulations, each containing varying proportions of *T. molitor* larvae powder (2, 4, and 6%). A comprehensive evaluation of key parameters, including fermentation characteristics, pH level, total acidity, specific volume, color characteristics, and sensory evaluations were performed, all of which were compared to a control bread, produced with conventional flour. The findings of this study revealed exciting insights into the impact of *T. molitor* larval flour on bread properties. Initially, as the concentration of insect flour in the bread increased, the amount of fermentation swelling decreased; this may be due to the fact that *T. molitor* larvae are considered gluten-free [83]. Along with the increase in insect flour in bread, the pH levels also increased, which may be due to the fact that the pH of the insect ranges from 6.4 to 7.5 [84]. As expected, with the increase in insect flour in bread, acidity levels decreased, which possibly resulted from the previous fact. A further interesting result is that the color characteristics of the bread sample remained almost unaffected, even though *T. molitor* larvae flour has a dark color owing to its composition. After a sensory evaluation (using a 9-point scale system), the bread with the addition of 2% *T. molitor* larvae flour recorded the highest scores in appearance, taste, and overall acceptability, indicating the promising potential of this flour as a protein-rich alternative ingredient in bakery products.

A few years later, breads were prepared with the addition of *T. molitor* larvae flour by Roncolini et al. [85]. Higher percentages of *T. molitor* larval flour, namely 5 and 10%, were incorporated in this later research. This research extended its focus to the analysis of the nutritional value of bread (protein, fat, EAAs, and EFAs) and the effect of the addition of *T. molitor* larval flour on the antimicrobial activity of bread products. According to the results, the bread with wheat flour contained 8.9% crude protein and as the addition of *T. molitor* larvae powder increased, the protein percentage also increased, finally reaching 11.6%, in the sample with 10% addition of insect flour, up to 30%. The same effects were shown for the fat content. In the control sample, bread with wheat flour, the fat percentage was 0.1%, while for the sample with 10% insect flour from *T. molitor* larvae, the fat percentage was 1.1%, showing a ten-fold increase. Finally, the ash content was also increased significantly from 0.5 to 0.6% in the above-mentioned samples. It is therefore shown that the addition of nutritional ingredients to conventional foods may enhance their nutritional value without being influenced by the preparation process, i.e., baking. In addition, the content of EAAs and EFAs was also studied, whereby by increasing the content of insect flour from *T. molitor* larvae, these important nutrients were also enhanced. Worth mentioning is the evaluation of the presence of endospores in the baked bread samples, along with the occurrence of the following human pathogens: *Clostridium perfringens* and *Bacillus cereus*. The occurrence of spores in all samples containing *T. molitor* flour was <1 while in the control sample was 0.38, and all prepared bread samples were subjected to enumeration of these two human pathogens and showed safe and viable counts below 1 log colony forming unit (CFU)/g [86]. Regarding sensory characteristics, color was evaluated where, unlike the previous study by Kim et al. [82] in which the color remained unaffected, the color of bread in this case progressively deepened with the increasing proportion of insect-derived flour. This observation likely stemmed from the notably higher percentages employed in this experiment compared to the previous study. Finally, in terms of consumption preference, white flour bread proved to be the most preferable, with the study conclusion being that, so far, the addition of more than 2% insect flour is not as preferred by consumers.

A expansion of the previous investigations was carried out by study Kuenper et al. [87], in which baked bread with a wider range of *T. molitor* larval flour concentrations, namely 0, 5, 10, and 15%, was examined. A combination of the characteristics studied in the previous studies was examined, leading to similar outcomes. In more detail, nutritional characteristics (protein, fat, carbohydrates, and fiber), antimicrobial activity, and consumer preference for the products were also explored in the present study. According to the results, increasing the content of *T. molitor* flour led to an increase in crude protein, fat, and fiber while simultaneously decreasing carbohydrates. The crude protein in bread made with conventional flour was recorded at 11.3 g while the highest value was recorded in bread with a 15% *T. molitor* larvae content, which was a 75.2% increase compared to the control sample. Fat increased from 2.2 to 4.8 g and fiber from 7.5 to 7.7 g, among the control sample and that containing 15% insect flour in both cases. Meanwhile, the carbohydrate content decreased as the content of *T. molitor* larval meal increased, which is expected since the carbohydrate content of insects is reduced due to their high crude protein content. Regarding the antimicrobial activity of the samples, the resistance of the bread to yeasts and molds was studied after 7 days. The results are highly promising, since no significant differences between the samples were found in terms of shelf life. Finally, according to the consumers, the bread with 5% *T. molitor* larval flour added was the most preferable despite being darker than the control one. This fact is greatly encouraging considering that, until now, consumers have not preferred the addition of insect flour to exceed 2%.

#### 3.1.2. Biscuits

Biscuits are the second most consumed bakery product after bread [88]. For this reason, *T. molitor* flour was also used to enhance these nutrient-poor products. The first attempt was made by Zielińska et al. [89] by preparing three types of biscuits enriched with *Τ. molitor* flour and the control biscuit with conventional flour. The control biscuit consisted of 300 g flour (M0), while the three versions receiving *T. molitor* flour contained 15 g (M15), 20 g (M20), and 30 g (M30), with the last sample being the darkest. As mentioned in the fortified bread, the higher the content of *T. molitor* flour, the higher the crude protein content of the biscuits. In M0, 9.1% crude protein content was recorded while in M30, it was 13.5%, i.e., a 48.3% increased content. However, the ash content was found to be greater in sample M20, with a value of 0.7%, while the highest antioxidant activity was found in sample M10, about 1 mM TE. Mixed results were obtained between the samples since the increase in *T. molitor* flour in the samples did not lead to a similar increase in nutrients in the biscuit samples. Nevertheless, it is worth noting that samples M15, M20, and M30 were significantly enriched compared to M0.

Biscuits with percentages of *T. molitor* larval flour, in order to enrich their nutritional value, were also prepared by Xie et al. [90]. Specifically, four biscuits with different percentages of *T. molitor* flour of 5% (M5), 10% (M10), 15% (M15), and 20% (M20) were prepared and compared with an insect-free control sample (M0). Concerning the nutritional value of the different biscuits in terms of protein, lipid, and dietary fiber, the higher the level of substitution of conventional flour with *T. molitor* flour, the higher the content of the above-mentioned nutrients in the samples. Sample M0 showed an equal crude protein content as in the aforementioned study, i.e., 9.1% [67], while the highest protein content was recorded in M20, which reached 16.0%. Moreover, the fat and dietary fiber content of M20 showed an increase of 20.5 and 21.7% compared to those of M0. Regarding organoleptic characteristics, the score for sensory properties showed no significant difference up to the 15% substitution level; however, in terms of preference, the biscuit with a 5% *T. molitor* flour content prevailed. Therefore, it was proven again that consumers do not prefer products containing over 5% *T. molitor* flour.

The latest study on the nutritional quality of biscuits enriched with *T. molitor* flour was conducted by Cozmuta et al. [91], where biscuits were prepared with 0, 10, 15, and 20% replacement of conventional flour with *T. molitor* flour. According to consumer preference, a significant result was recorded as the biscuit enriched with 15% *T. molitor* flour was selected as the most preferred biscuit, and it was the first time that consumers showed preference for such a high percentage of *Τ. molitor* flour in a food product. Comparing the conventional biscuit with the fortified biscuit, the nutritional value was significantly increased. Specifically, crude protein increased from 29.1 to 31.3%, crude fat increased from 46.4 to 47.8%, and ash increased from 1.4 to 1.6%. In addition, all individual minerals were identified and quantified, where in particular the addition of 15% *T. molitor* flour resulted in enhanced nutritional value in mineral elements. In detail, Na, K, Ca, Mg, P, Cu, Zn, Mn, Fe and Li showed an increase in content of about 2.1, about 7.1, 6.4, 20.6, 0.1, 39.5, 50.1, 13.2, 25.3 and 13.3%, respectively, proving once again that *T. molitor* larval flour can contribute to enhancing otherwise-nutrient-poor food products, such as bread and biscuits [92].

#### 3.1.3. Cracker

**Figure 1:**
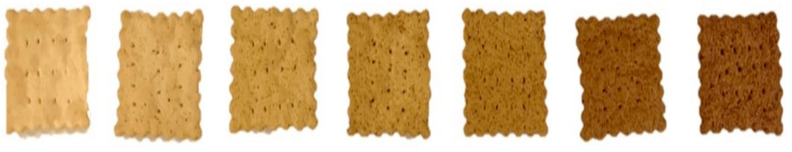
Crackers containing 0 (control) to 20% *T. molitor* flour (% *w*/*w*) from (**right**) to (**left**) [93]. Reprinted/adapted with permission from Ref. [93]. 2022, MDPI.

Further study of the addition of *Τ. molitor* larvae flour to bread has not been carried out and, thus, the first attempt to enrich another amylaceous product, cracker, was made by Djouadi et al. [93]. In this study, crackers incorporating varying proportions of *T. molitor* larvae flour (0, 2, 4, 6, 10, 15, and 20%) were prepared (see Figure 1), where 0% was used as a control sample as in previous studies. The color of the crackers darkened greatly as the *T. molitor* larvae flour content increased, and therefore, consumers showed no attraction to crackers containing 10% or more *T. molitor* larvae flour. Hence, the researchers evaluated only the cracker with 6% insect flour and compared it to the control cracker. Regarding the nutritional value of the two crackers, 0 and 6% insect flour, the protein content scored values from 9.7 to 13.9%, respectively, and the ash content of the crackers scored values from 1.9 to 2.2%. Moreover, besides the increase in total ash content, the content of constituent minerals such as Na, K, Ca, Mg, P, Fe, Cu, and Zn in the cracker with 6% *T. molitor* flour increased strongly. Delving deeper into the study of the nutritional value of the 6% cracker, it was found that its polyphenol content also showed a statistically significant (*p* < 0.05) increase compared to the control sample, which was enhanced by 148.8%. Polyphenols are known for their strong antioxidant properties [94]. Therefore, it is believed that the high polyphenol content may have enhanced the antioxidant activity of the cracker, since through this study using two different methods (2,2-diphenyl-1-picrylhydrazyl (DPPH) radical scavenging activity and the reducing power of ferric ion), the cracker with the *T. molitor* larvae flour showed a significantly higher antioxidant activity than the control sample.

#### 3.1.4. Bars

An investigation in which whole *T. molitor* larvae were used to produce a snack (bars) was carried out in Poland [95]. The research team developed four kinds of bars: bars in the absence of edible insects (serving as control), bars containing 7.4% *w*/*w* whole mealworms (they were visible to consumers), bars containing 7.4% *w*/*w* ground mealworms, and bars containing 7.4% *w*/*w* ground crickets. This study was conducted to assess consumer acceptance of snack bars enriched with edible insects and whether preference is influenced by the appearance of a food rather than its composition.

The findings of this study revealed intriguing consumer preferences. Notably, the control bars, which served as the control, were favored over the ground insect-enriched counterparts. Moreover, participants noted that the appearance of bars containing crickets was less appealing compared to the other products, highlighting the significance of visual appeal in consumer choices. The study underscored that deliciousness emerged as the primary determinant factor of the acceptability of insect-infused bars as a food source. According to the results, the bar with *T. molitor* larvae flour was much more preferable than the one with whole larvae, and in fact, the study underlines that in terms of appearance and taste, the control bars and those containing non-visibly embedded *T. molitor* larvae do not show significant differences. Additionally, the odor of the product emerged as a statistically significant indicator of quality, further emphasizing the importance of sensory aspects in consumer preferences. Considering the results from the odor of the samples, the control sample had the most attractive odor, followed by the sample with no obvious edible insects and the sample with obvious edible insects. As far as the samples with *T. molitor* larvae were concerned, the appearance of the larvae seemed to have influenced the odor of the food product to a large extent. Collectively, this research highlights that in terms of appearance and taste, control bars and those containing non-visibly integrated edible insects do not exhibit significant differences. This suggests that it is the phenomenon of neophobia, the reluctance to embrace new or unfamiliar foods like insects, that instills suspicion in people regarding insect consumption. These findings are invaluable in understanding the nuanced factors that influence consumer acceptance of insect-based food products. The proximate composition of the different baked products containing *T. molitor* flour is shown in Table 4 below.

### 3.2. Meat

#### 3.2.1. Burgers

*T. molitor,* aside from the bakery products, was also employed in meat-based products. To this end, distinct burger formulations were prepared at the University of Liège [96]. Specifically, a beef burger consisting of unflavored ground beef, a lentil-based variant (consisting of 95% green lentils), a burger with 45% green lentils and 50% *T. molitor*, and a burger containing 45% ground beef and 50% *T. molitor* were produced. In all cases, a 5% fraction of each burger consisted of a flavoring mix (containing onions, carrots, tomato paste, garlic, salt and pepper). These burgers were prepared in order to examine the degree of insect neophobia in Western societies. In the analysis of the participants’ ratings, the gender of the participant was found to play a significant role. As far as the taste of the burger was concerned, all participants favored the traditional beef burger, ranking it first. However, it is worth noting that the male participants preferred burger preparations containing beef or *T. molitor* more than the fully vegetable ones. At the same time, women showed a greater preference for conventional beef burgers only. In addition, odor did not seem to be significantly affected by the composition of the samples according to both genders. One notable outcome was the positive inclination observed among the participants regarding the integration of insects into their future dietary habits. Impressively, 70% of the respondents expressed confidence in the prospective inclusion of insects within their diets.

#### 3.2.2. Sausages

Kim et al. [97], being the first researchers to do so, decided to prepare emulsion sausages which also contained various types of *T. molitor* flour. Specifically, they prepared a conventional pork sausage containing 60% lean pork, 20% fat, and 20% ice (control sausage). At the same time, those containing insects were prepared by replacing 10% of the lean pork with flour of *T. molitor* larvae. Essentially, three different insect sausages were prepared, since three different *T. molitor* flours were used. Specifically, untreated *T. molitor* larvae flour, defatted *T. molitor* larvae flour, and defatted and acid-hydrolyzed *T. molitor* larvae flour were employed. Results indicated that the increased treatment of *T. molitor* flour resulted in an increased protein content of the final sample. In other words, a higher percentage of proteins was found in the sausage with defatted and acid-hydrolyzed flour from *T. molitor* larvae; the value increased from 22.6 to 31.3%. In addition to protein, minerals contained in the final samples were also evaluated. The results of these showed varying results. The addition of *T. molitor* larval flour, regardless of the treatment it received, increased the content of all the minerals tested (P, K, Ca, Mg, Na, Zn, Fe, Cu, and Mn). Nevertheless, the sample containing defatted and acid-hydrolyzed flour from *T. molitor* larvae presented the lowest mineral content compared to the other two samples containing *T. molitor*. The highest content of Ca (13.9 mg/100 g), Na (913.7 mg/100 g), Fe (9.9 mg/100 g), Cu (0.3 mg/100 g), and Mn (0.4 mg/100 g) was recorded in the sample with 10% *T. molitor* larvae flour that no additional treatment was applied to. Simultaneously, a higher content of P (202.1 mg/100 g), K (325.2 mg/100 g), Mg (47.7 mg/100 g), and Zn (2.5 mg/100 g) was recorded in the sample with 10% defatted *T. molitor* larvae flour. At this point it should be noted that the control sample showed the following mineral contents (P—145.5 mg/100 g, K—245.7 mg/100 g, Ca—9.3 mg/100 g, Mg—20.9 mg/100 g, Na—799.5 mg/100 g, Zn—1.2 mg/100 g, Fe—3.8 mg/100 g, Cu—0.04 mg/100 g and Mn—0.1 mg/100 g). Thus, it is proven that a nutrient product can be enhanced even with the addition of 10% of insect flour. Furthermore, it is proven that the addition of *T. molitor* larvae flour to a food product can achieve different outcomes depending on the treatment it has received. Hence, depending on the nutrients insisted upon to be enhanced in a product, the flour used should receive a corresponding treatment.

Frankfurt sausages are known worldwide due to their inclusion in the well-known product “hot dog” [98]. Choi et al. [99] attempted to enrich this sausage with insect flour in various proportions in order to evaluate its nutritional value and consumer preference. This substitution with insect flour is also carried out in order to reduce the negative environmental footprint caused by the production of the pork [8,100] from which the Frankfurt sausage is made. As control samples, the sausages were prepared with 50% pork ham, 25% back fat, and 25% ice. The other samples were prepared as follows: T1, 45% pork ham + 5% *T. molitor* larvae flour; T3, 35% pork ham + 15% *T. molitor* larvae flour; T4, 30% pork ham + 20% *T. molitor* larvae flour; T5, 25% pork ham + 25% *T. molitor* larvae flour; and T6, 20% pork ham + 30% *T. molitor* larvae flour. The composition of back fat and ice in each treatment was not different from the control. As occurred in previous studies, increasing the amount of *T. molitor* flour in the samples resulted in an enhanced protein content of the samples. Specifically, the crude protein content was 10.0% in the control sample, 13.9% in T1, 14.3% in T2, 15.6% in T3, 17.5% in T4, 21.1% in T5 and 24.0% in T6. Likewise, a similar increase was observed in the total mineral content (ash) as the content was recorded at 2.1% in the control sample, 2.3% in T1, 2.3% in T2, 2.3% in T3, 2.6% in T4, and about 2.9% in T5 and T6. Hence, the use of *T. molitor* larvae flour is not necessarily limited to the preparation of bakery products, since it may also enrich the nutritional value of further foodstuffs. According to the sensory evaluation, sausages with *T. molitor* were poorer in color, flavor, off-flavor, and juiciness scores. However, consumers indicated that up to the T2 sample, there was no noticeable difference from the control sample. Consequently, another widely consumed food can be enriched and rendered eco-friendlier without affecting consumer preferences.

### 3.3. Sauces

In Asia, one of the main protein foods is soy sauce [101], which apart from its high crude protein content, contains all the EAAs such as His, Met, which imparts the bitter flavor and Lys, which imparts the sweetness [102,103]. In an attempt to enhance the nutritional value of this sauce, six different variations of the sauce were prepared by Cho et al. [104]. Specifically, the samples were as follows: S8: soy sauce with soybean meju:koji:roasted rice flour = 8:1:1; S6: soy sauce with soybean meju:koji:roasted rice flour = 6:2:2; M8: raw insect sauce with mealworm meju:koji:roasted rice flour = 8:1:1; M6: raw insect sauce with mealworm meju:koji:roasted rice flour = 6:2:2; DM8: defatted insect sauce with defatted mealworm meju:koji:roasted rice flour = 8:1:1; DM6: defatted insect sauce with defatted mealworm meju:koji:roasted rice flour = 6:2:2; and conventional soy sauce. The fermentation period extended to 20 days at a controlled temperature of 25 °C. After the nutritional value assessment, consumer acceptance was examined. With respect to the results of EAAs, the composition of the soy sauce seemed to influence their quantity in a different way than presented in previous studies. This means that the addition of *T. molitor* larvae to the samples did not seem to enhance the content of all EAAs. To illustrate, the arginine content was found to be highest in samples S6 (225.4 mg/100 g) and S8 (100.8 mg/100 g) and the Phe content was highest in sample S6 (292.0 mg/100 g). Concerning the other EAAs, the addition of *T. molitor* larvae greatly favored their content, with sample M6 leading the way. Moreover, the defatting of the samples seemed to have negatively affected the *T. molitor* samples, possibly due to the longer series of experimental treatments the larvae received. Thereby, it was concluded that *T. molitor* larvae can be added to a range of foods, which can be either in solid or liquid form. However, it is worth mentioning that the total nitrogen content, a critical parameter, in samples S8, DM6, and DM8 ranged between 1.06 and 1.19% after 20 days of fermentation, exceeding the reference limit of 0.70% total nitrogen set by the FAO/WHO *Codex Alimentarius* international standard for soy sauce. Considering some organoleptic characteristics, it was reported that sauces with *T. molitor* larvae had a yellowish color but pH items did not seem to be influenced by the composition of the sauce. Of great interest was that consumer preference for the samples was studied on a comprehensive 7-point scale and showed no significant differences between the insect and soy sauce. Consequently, insect sauce appeared similar to soy sauce, confirming its potential to be marketed in liquid spice form. This mitigates consumer rejection due to its shape and texture characteristics, ensuring wider acceptance of this product.

Due to the popularity of soy sauce, a second attempt was made to produce enriched soy sauce with flour from *T. molitor* larvae. Specifically, Lee and Kim [105] used four different combinations of materials to prepare the soy sauces. The materials used were ground soybean, mixed ground soybean with heated okara, defatted *T. molitor* larvae powder, and both heated okara and defatted *T. molitor* larvae. From these materials, five sauces were prepared: one high-salt soy sauce with ground soybean (sample 1), one low-salt soy sauce with ground soybean (sample 2), one low-salt soy sauce with mixed ground soybean with heated okara (sample 3), one low-salt soy sauce with defatted *T. molitor* larvae powder (sample 4), and finally, one low-salt soy sauce with both heated okara and defatted *T. molitor* larvae (sample 5). The sauces were analyzed for their content of free amino acids, their antioxidant capacity, and their sensory characteristics. Regarding the different free amino acids, all 20 [106] were detected in the soy sauces, with sample 4 having the highest content of free amino acids (28.6 mg/L), followed by samples 5, 1, 2, and 3 with contents of 25.7, 11.5, 9.6, and 2.1 mg/L, respectively. Furthermore, with regard to amino acids, the most abundant amino acid in these soy sauces was glutamic acid (593–5600 mg/L), with samples 4 and 5 recording the highest values of 4830 and 5600 mg/L, respectively. Therefore, it has been initially confirmed that the enrichment of existing foods with *T. molitor* flour enhances their nutritional value, and more specifically, the addition of nymph powder increased the glutamic acid content through fermentation, as previously reported by Yoo et al. [107]. In addition to the high content of amino acids, samples 4 and 5 also showed a significant increase in their antioxidant capacity. Finally, perhaps the most significant result is the preference of consumers for the sauces. Sample 5 seems to have made the strongest impression among the other samples, as it ranked first in terms of taste and overall preference. Therefore, for the second time, it has been demonstrated that flour from *T. molitor* larvae is suitable for producing more nutritionally enriched soy sauces, with a low salt content, in fact.

### 3.4. Dairy Products

#### 3.4.1. Ice Cream

Ice cream is a frozen dairy food product consisting of cream or butter fat, milk, sugar, and flavors [108,109]. As a favorite dessert for all age groups of people of all genders, hundreds of flavors have been invented, with vanilla, chocolate, and strawberry being the most popular. Ice creams are considered fattening desserts with no particular nutritional value; hence, an effort was made to enrich the nutritional value of ice cream [110]. To this end, a distinctive product—strawberry–cranberry ice cream containing *T. molitor* larvae, was developed [111]. In more detail, four products were developed, including a control sample (HCOo). The first was with the addition of *T. molitor* larvae (HΤ), the second with a mixture of *T. molitor* larvae and chia (HTC), and the last with the addition of a mixture of larvae and quinoa (HTQ). The ingredients were weighed, mixed, and pasteurized at 68 °C for 30 min. The addition of the strawberry–cranberry pulp, seeds, and larvae necessitated an aging process at 4 °C for 24 h, after which the mixture was beaten for 10 min and frozen at −4 °C. The addition of an insect led to an increase in crude protein, vitamins, and minerals in the product, resulting in a significant increase in the nutritional value of the ice cream. In detail, the highest percentage of protein was found in the HT sample, 11.8%, and the following protein richest ice cream was the HTC sample, 11.4%, i.e., 26.2 and 21.4% higher than the control sample, respectively. The most nutritionally enhanced samples were HT and HTC, with the latter showing the highest nutritional value. Namely, the content of Ca, P, Na, K, Mg, and Fe in HTC was 332.0, 224.5, 232.8, 408.2, 50.0, and 1.6 mg/100 g, respectively, while the Ca, P, Na, K, Mg, and Fe content of HT was 265.4, 185.1, 215.3, 403.9, 32.1, and 1.5 mg/100 g, respectively. In addition to the increase in minerals, there was a great increase in vitamins, where the contents of vitamins A, D, E, K B1, B2, B3, B9, and B12 in HTC were 1.9, 949.2, 9.7, 997.0, 27.3, 33.4, 102.9, 40.0 and 4.7 mg/100 g, respectively. At the same time, the contents of HTC in vitamins A, D, E, K B1, B2, B3, B9, B12 were 1.0, 584.3, 5.9, 0.0, 22.6, 33.7, 86.7, 36.8 and 4.4 mg/100 g, respectively, while the contents of the same vitamins in the control sample were 0.9, 479.7, 4.8, 0.0, 21.5, 30.6, 74.6, 17.5 and 4.4 mg/100 g, respectively. A further application of *T. molitor* larvae flour is demonstrated in the present research, paving the way for other foods in which *T. molitor* flour can be incorporated and enhance its nutritional value.

#### 3.4.2. Milk

Milk is a common household food product with unique functional, nutritional, and sensory properties [112,113]. Recent research from Maastricht University confirmed that the protein of *T. molitor* larvae matches the gold standard of milk protein due to its complete profile of EAAs and digestion [27], and for this reason, Tello et al. [114] decided to prepare an innovative product, i.e., milk from *T. molitor* larvae, crafted to mitigate the environmental impact associated with conventional bovine’s milk production. The insect milk created consisted of 1.19% crude protein, 5.76% fat, and <1% carbohydrate. The impact of the insect milk prototype was 0.76 kg CO_2_ equivalents per kg FPCM in global warming potential, while bovine milk accounts for 1.6 gigatons CO_2_ equivalents per year [115]. However, this milk is in a pilot stage and individual processes need to be implemented in order to prepare a final product and make it edible and probably marketable. Nevertheless, it has paved the way for a still-widely consumable product that will greatly reduce the negative impact of bovine milk production.

#### 3.4.3. Spreadable Cheese

**Figure 2:**
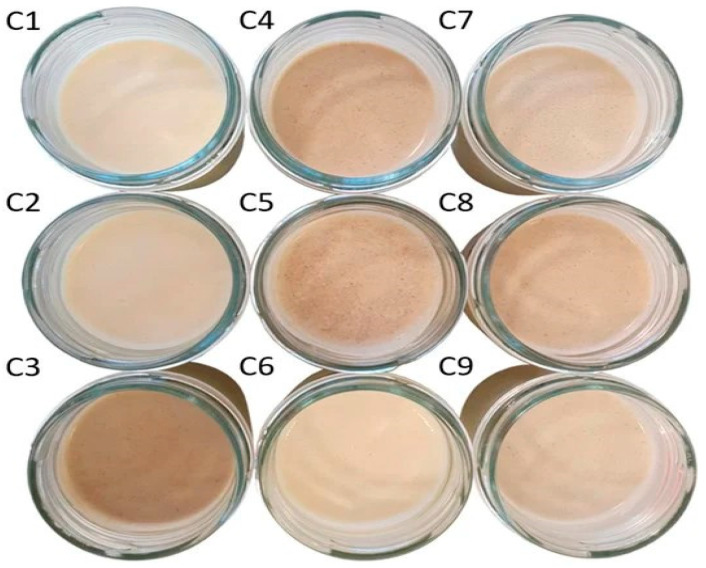
Image of the nine spreadable cheese analogues used in the experiments, labeled from C1 to C9 [116]. Reprinted/adapted with permission from Ref. [116]. 2023, MDPI.

The most recent research conducted for the preparation of enriched food with *T. molitor* larvae was carried out in the current year, 2023. A hybrid product of spreadable cheese was produced using nine different combinations of three different ingredients: milk protein concentrate, faba bean flours, and *T. molitor* flour, which together constituted 7.1% of the formula [116]. The samples had the following proportions of the ingredients: milk protein concentrate, faba bean flours, and *T. molitor* flour—100/0/0 (control sample)—C1, 50/50/0—C2, 0/50/50—C3, 50/0/50—C4, 75/0/25—C5, 75/25/0—C6, 25/50/25—C7, 25/25/50—C8, and 50/25/25—C9 (see Figure 2).

These nine samples were evaluated both in terms of their nutritional value and their sensory characteristics. Regarding the protein profile of the samples, it appears that the addition of two protein ingredients, *T. molitor* flour and faba bean flour [117], did not enhance the protein content of the spreadable cheese analogues. Specifically, the protein contents recorded in samples C1–C9 were 5.2%, 4.8%, 4.2%, 4.6%, 4.9%, 5.0%, 4.5%, 4.4%, and 4.7%, respectively. Additionally, it was evident that the immediately higher protein value was recorded in sample C6, 3.9% lower than the control sample. As expected, the sample with the highest protein content also had the lowest fat content. Specifically, sample C1 (high protein sample) had a fat content of 30.1%, while sample C3 (low protein sample) had a fat content of 31.9%. Furthermore, in terms of sensory characteristics, samples C2, C7, and C9 had the best flavor as they exhibited a more characteristic cheesy taste compared to the other samples. The most promising result is that the samples containing *T. molitor* flour showed a more preferable flavor profile compared to the samples that did not contain *T. molitor* flour.

### 3.5. Oil

A survey on the suitability of *T. molitor* larvae oil for consumption or as an ingredient in various foods was carried out by Son et al. [118]. The oil from larvae was presented as a light-yellow liquid, like commonly used edible oils. The peroxide value of commercialized edible oils was estimated at less than 2 mequivalents/kg oil [119] while *T. molitor* oil showed a much higher value, of the order of 3.5 mequivalents/kg oil. In order to examine the samples for their stability, secondary oxidation markers (TBARS) were determined. The lower the value of TBARS, the higher the stability of the oils [120], and in this case the oil showed a value of 1.8 mg MDA/kg oil. Furthermore, the tocopherol (vitamin E) content of the oil was studied, which is a natural antioxidant that prevents lipid oxidation by free radicals [121]. In terms of animal food sources, chicken and pork contained 2.7–4.2 and 9.5–100.0 mg/kg of total vitamin E, respectively [122,123]. Meanwhile, olive oil and soybean oil ranged from 80.0 to 1360.0 mg/kg of vitamin E [124]. In accordance with the present study, the oil from *T. molitor* larvae was rich in vitamin E, with a content of 144.3 mg/kg oil; therefore, it was concluded that it can be easily compared with the main consumed oils. Rigorous safety assessments, including a comprehensive toxicological profile on rats over 28 days, revealed significant reductions in cholesterol and glucose levels without adverse hematological effects or mortality. This unequivocally establishes the safety and nutritional superiority of insect oils. Intriguingly, after a one-year storage period, the oil was found to be stable, highlighting its potential as a novel food ingredient suitable for commercial distribution.

### 3.6. Tenebrio molitor as Animal Feed to Enhance Meat for Human Consumption

In addition to the production of food products fortified with *T. molitor* larvae flour, a further way of consuming insect-fortified conventional foods exist. In particular, this alternative route for consuming insects exists indirectly through the consumption of meat or fish raised either exclusively or for a large part of their lives with insects. One such case is the study by Gasco et al. [125], in which they bred rabbits subjected to diets enriched with fat from *T. molitor* and other insects to ascertain the advantages that a rabbit diet can provide on the final nutritional value. Three samples were tested: the control sample and rabbits fed with a diet containing 50% fat from *T. molitor* and rabbits fed with a diet containing 100% fat from *T. molitor*. The final product was examined for its crude protein content and this revealed that all the samples had the same protein content. In addition, the EFA content was not affected by the diet of the samples. Nevertheless, the rabbit meat fed with diets containing *T. molitor* fat was less susceptible to oxidation. Moreover, a sensory test was carried out by young individuals who consumed meat at least twice a week. According to their preferences, they did not perceive any difference between the rabbit meats, which means that organoleptically there would be no difference between the ‘classic’ meats and the meat that was fed with insects. However, the meat of rabbits reared with insects was not nutritionally enhanced so it cannot be considered enriched or extra beneficial for consumption.

## 4. Conclusions and Future Perspectives

*T. molitor* larvae are increasingly gaining ground in the food sector as they are no longer studied only for their nutritional value and their value as animal food, but also ways of introducing them into the human diet are being investigated. A growing amount of research is being carried out in order to develop new tasty and nutritious products prepared with *T. molitor* larvae, especially in bakery products or products containing flour, such as burgers. There are three reasons why flour is the main product of *T. molitor* larvae used for food preparation. Firstly, the production of flour from *T. molitor* larvae is exceptionally simple and economical; secondly, the flour can be added to a variety of foods, increasing their nutritional characteristics (proteins, EAAs, EFAs, and vitamins); and finally, they are largely accepted in foods by consumers. This means that neophobia in Europe is slowly disappearing in terms of insect consumption and people are starting to accept insects as a new and innovative food, either directly or indirectly, given that the use of insects as animal feed has been ongoing for years. This fact is very encouraging as new enhanced nutritional foods can now be produced to promote a more sustainable way of production. These foodstuffs are not required to be completely novel, since the enhancement of conventional foodstuffs with flour from *T. molitor* larvae has been proven to result in new and advanced products. These products may differ in color from conventional ones, as mentioned above. Still, once a preferable rate of insect flour addition/replacement by consumers has been established, products can be created more efficiently. Finally, as stated above, flour is not the only product that can be used containing *T. molitor* larvae, as it turned out that oil containing them can also be a safe and nutritional choice either for direct consumption or as an additive or substitute for oils in various food products. Moreover, the oil is an undetectable ingredient in food, which will promote rapid consumer acceptance. Overall, the development of food products that are either based on or enhanced with *T. molitor* larvae has begun, and the current studies are paving the way for new avenues to be explored, so as to revolutionize the food market. 

## Figures and Tables

**Table 1:** Nutritional value of *T. molitor* larvae according to the crude protein quantification method.

Composition of Dry Weight (%)			
Crude Protein	Crude Fat	Ash	Ref.
36.8	26.0	~1.0	[60]
46.4	32.2	2.9	[35]
47.0	29.6	2.6	[56]
51.0	ne *	ne	[59]
60.2	19.1	4.2	[58]
75.1	ne	ne	[61]

* ne: not examined.

**Table 2:** Amino acid composition (mg/g of dry weight) of *T. molitor* larvae.

His	Iso	Leu	Lys	Met	Phe	Thr	Trp	Val	Ref.
0.8	1.3	2.2	1.6	0.6	1.3	1.3	0.3	2.2	[67]
1.5	3.6	3.4	2.9	0.7	1.6	1.8	nd *	2.4	[57]
2.6	2.8	4.8	1.8	1.4	1.4	2.9	1.9	4.0	[43]
2.8	6.5	6.2	5.3	1.2	3.2	3.3	0.02	4.5	[68]
3.1	4.0	7.3	5.8	2.2	1.8	4.3	0.7	5.3	[69]

* nd: not detected; His: histidine; Iso: isoleucine; Leu: leucine; Lys: lysine; Met: methionine; Phe: phenylalanine; Thr: threonine; Trp: tryptophane; Val: valine.

**Table 3:** Fatty acid composition (%) of *T. molitor* larvae.

C14:0	C16:0	C16:1	C18:1	C18:2 (ω-6)	C20:0	C18:3 (ω-3)	∑ SFA	∑ UFA	Ref.
2.1	18.8	0.5	28.6	48.1	0.6	0.2	22.5	77.5	[58]
2.1	17.2	1.9	43.8	29.4	nr *	2.3	21.0	79.0	[62]
2.3	21.4	0.1	39.1	27.3	1.4	0.1	31.6	nr	[65]
4.3	21.1	1.9	52.9	11.5	0.5	0.2	33.2	nr	[66]
5.0	19.1	1.7	49.9	18.1	0.1	0.4	28.6	nr	[67]

* nr: not referred.

**Table 4:** Proximate composition of the various bakery products made with the addition of *T. molitor* flour.

Bakery Products	Percentage (%) of *T. molitor* Flour Addition	Protein Content (g/100 g)	Fat Content (g/100 g)	Ash Content (g/100 g)	Ref.
Bread	0	8.9	0.1	0.5	[85]
	5	10.5	0.5	0.5	
	10	11.6	1.1	0.6	
	0	9.6	ne *	ne	[87]
	5	12.6	ne	ne	
	10	13.2	ne	ne	
	15	13.7	ne	ne	
Biscuits	0	9.1	27.0	0.3	[89]
	15	13.5	27.2	0.6	
	20	11.9	26.9	0.7	
	30	10.8	28.5	0.4	
	0	9.1	18.5	2.0	[90]
	5	10.3	19.4	2.1	
	10	13.0	19.7	2.3	
	15	14.2	20.5	2.3	
	20	16.0	22.3	2.4	
	0	29.1	46.4	1.4	[91]
	15	31.3	47.8	1.6	
Cracker	0	9.7	12.7	1.9	[93]
	6	13.9	11.1	2.2	

* ne: not examined.

## Data Availability

Data is contained within the article.

## References

[1] Wallace B. (1997). The Multidimensional Nature of Population/Environmental Problems. Polit. Life Sci..

[2] Béné C., Barange M., Subasinghe R., Pinstrup-Andersen P., Merino G., Hemre G.I., Williams M. (2015). Feeding 9 Billion by 2050–Putting Fish Back on the Menu. Food Secur..

[3] Boyd C.E., McNevin A.A., Davis R.P. (2022). The Contribution of Fisheries and Aquaculture to the Global Protein Supply. Food Secur..

[4] Van Bavel J. (2013). The World Population Explosion: Causes, Backgrounds and -Projections for the Future. Facts Views Vis. ObGyn.

[5] Henchion M., Hayes M., Mullen A.M., Fenelon M., Tiwari B. (2017). Future Protein Supply and Demand: Strategies and Factors Influencing a Sustainable Equilibrium. Foods.

[6] Foley J.A., Ramankutty N., Brauman K.A., Cassidy E.S., Gerber J.S., Johnston M., Mueller N.D., O’Connell C., Ray D.K., West P.C. (2011). Solutions for a Cultivated Planet. Nature.

[7] Le Mouël C., Forslund A. (2017). How Can We Feed the World in 2050? A Review of the Responses from Global Scenario Studies. Eur. Rev. Agric. Econ..

[8] Caro D. (2018). Greenhouse Gas and Livestock Emissions and Climate Change.

[9] Van Der Zijpp A., Wilke P., Carsan S. (2008). Sustainable Livestock Intensification the Role of Livestock in Developing Communities: Enhancing Multifunctionality.

[10] Copping A.E., Hemery L.G., Viehman H., Seitz A.C., Staines G.J., Hasselman D.J. (2021). Are Fish in Danger? A Review of Environmental Effects of Marine Renewable Energy on Fishes. Biol. Conserv..

[11] Dulvy N.K., Pacoureau N., Rigby C.L., Pollom R.A., Jabado R.W., Ebert D.A., Finucci B., Pollock C.M., Cheok J., Derrick D.H. (2021). Overfishing Drives over One-Third of All Sharks and Rays toward a Global Extinction Crisis. Curr. Biol..

[12] Coleman F.C., Williams S.L. (2002). Overexploiting Marine Ecosystem Engineers: Potential Consequences for Biodiversity. Trends Ecol. Evol..

[13] Lachs L., Oñate-Casado J. (2020). Fisheries and Tourism: Social, Economic, and Ecological Trade-Offs in Coral Reef Systems.

[14] Patel S., Suleria H.A.R., Rauf A. (2019). Edible Insects as Innovative Foods: Nutritional and Functional Assessments. Trends Food Sci. Technol..

[15] Egas-Montenegro E., Ordo R. (2021). International Journal of Gastronomy and Food Science Edible Insects: A Food Alternative for the Sustainable Development of the Planet. Int. J. Gastron. Food Sci..

[16] Guin R.P.F., Florença S.G., Boustani N.M., Chuck-hern C., Sari M.M., Ferreira M., Costa C.A., Cardoso A.P., Tarcea M., Correia P.M.R. (2022). Are Consumers Aware of Sustainability Aspects Related to Edible Insects? Results from a Study Involving 14 Countries. Sustainability.

[17] Florença S.G., Guin R.P.F., Gonçalves F.J.A., Jo M., Ferreira M., Costa C.A., Correia P.M.R., Cardoso A.P., Campos S. (2022). The Motivations for Consumption of Edible Insects: A Systematic Review. Foods.

[18] Ha N.I., Mun S.K., Im S.B., Jang H.Y., Jeong H.G., Kang K.Y., Park K.W., Seo K.S., Ban S.E., Kim K.J. (2022). Changes in Functionality of *Tenebrio molitor* Larvae Fermented by *Cordyceps militaris* Mycelia. Foods.

[19] Premalatha M., Abbasi T., Abbasi T., Abbasi S.A. (2011). Energy-Efficient Food Production to Reduce Global Warming and Ecodegradation: The Use of Edible Insects. Renew. Sustain. Energy Rev..

[20] Krongdang S., Phokasem P., Venkatachalam K., Charoenphun N. (2023). Edible Insects in Thailand: An Overview of Status, Properties, Processing, and Utilization in the Food Industry. Foods.

[21] Siddiqui S.A., Shah M.A., Centoducati G. (2023). Prospects of Edible Insects as Sustainable Protein for Food and Feed—A Review. J. Insects Food Feed..

[22] van Huis A. (2015). Edible Insects Contributing to Food Security?. Agric. Food Secur..

[23] Van Huis A. (2016). Edible Insects Are the Future?. Proc. Nutr. Soc..

[24] Kouřimská L., Adámková A. (2016). Nutritional and Sensory Quality of Edible Insects. NFS J..

[25] Ojeda-Avila T., Woods H.A., Raguso R.A. (2003). Effects of Dietary Variation on Growth, Composition, and Maturation of *Manduca sexta* (Sphingidae: Lepidoptera). J. Insect Physiol..

[26] Bjørge J.D., Overgaard J., Malte H., Gianotten N., Heckmann L.H. (2018). Role of Temperature on Growth and Metabolic Rate in the Tenebrionid Beetles *Alphitobius Diaperinus* and *Tenebrio molitor*. J. Insect Physiol..

[27] Mancini S., Moruzzo R., Riccioli F., Paci G. (2019). European Consumers’ Readiness to Adopt Insects as Food. A Review. Food Res. Int..

[28] Piha S., Pohjanheimo T., Lähteenmäki-Uutela A., Křečková Z., Otterbring T. (2018). The Effects of Consumer Knowledge on the Willingness to Buy Insect Food: An Exploratory Cross-Regional Study in Northern and Central Europe. Food Qual. Prefer..

[29] Moruzzo R., Mancini S., Boncinelli F., Riccioli F. (2021). Exploring the Acceptance of Entomophagy: A Survey of Italian Consumers. Insects.

[30] van Huis A., Rumpold B. (2023). Strategies to Convince Consumers to Eat Insects? A Review. Food Qual. Prefer..

[31] Żuk-Gołaszewska K., Gałęcki R., Obremski K., Smetana S., Figiel S., Gołaszewski J. (2022). Edible Insect Farming in the Context of the EU Regulations and Marketing—An Overview. Insects.

[32] (2021). Commission Implementing Regulation (EU) 2021/1975 of 12 November 2021 Authorising the Placing on the Market of Frozen, Dried and Powder Forms of *Locusta Migratoria* as a Novel Food under Regulation (EU) 2015/2283 of the European Parliament and of the Council and Amending Commission Implementing Regulation (EU) 2017/2470. Off. J. Eur. Union L402.

[33] (2021). Commission Implementing Regulation (EU) 2021/882 of 1 June 2021 Authorising the Placing on the Market of Dried *Tenebrio molitor* Larva as a Novel Food under Regulation (EU) 2015/2283 of the European Parliament and of the Council, and Amending Commission Implementing Regulation (EU) 2017/2470. Off. J. Eur. Union L194.

[34] (2023). Commission Implementing Regulation (EU) 2023/58 of 5 January 2023 Authorising the Placing on the Market of the Frozen, Paste, Dried and Powder Forms of *Alphitobius Diaperinus* Larvae (Lesser Mealworm) as a Novel Food and Amending Implementing Regulation (EU) 2017/2470. Off. J. Eur. Union L5.

[35] Moruzzo R., Riccioli F., Espinosa Diaz S., Secci C., Poli G., Mancini S. (2021). Mealworm (*Tenebrio molitor*): Potential and Challenges to Promote Circular Economy. Animals.

[36] Bordiean A., Krzyżaniak M., Stolarski M.J., Czachorowski S., Peni D. (2020). Will Yellow Mealworm Become a Source of Safe Proteins for Europe?. Agriculture.

[37] Tzompa-Sosa D.A., Moruzzo R., Mancini S., Schouteten J.J., Liu A., Li J., Sogari G. (2023). Consumers’ Acceptance toward Whole and Processed Mealworms: A Cross-Country Study in Belgium, China, Italy, Mexico, and the US. PLoS ONE.

[38] Gkinali A.-A., Matsakidou A., Vasileiou E., Paraskevopoulou A. (2022). Potentiality of *Tenebrio molitor* Larva-Based Ingredients for the Food Industry: A Review. Trends Food Sci. Technol..

[39] Fischer A.R.H., Steenbekkers L.P.A. (2018). All Insects Are Equal, but Some Insects Are More Equal than Others. Br. Food J..

[40] Mancuso T., Pippinato L., Gasco L. (2019). The European Insects Sector and Its Role in the Provision of Green Proteins in Feed Supply. Qual. Access Success.

[41] Sogari G., Amato M., Biasato I., Chiesa S., Gasco L. (2019). The Potential Role of Insects as Feed: A Multi-Perspective Review. Animals.

[42] Thévenot A., Rivera J.L., Wilfart A., Maillard F., Hassouna M., Senga-Kiesse T., Le Féon S., Aubin J. (2018). Mealworm Meal for Animal Feed: Environmental Assessment and Sensitivity Analysis to Guide Future Prospects. J. Clean. Prod..

[43] Selaledi L., Mbajiorgu C.A., Mabelebele M. (2020). The Use of Yellow Mealworm (*T. Molitor*) as Alternative Source of Protein in Poultry Diets: A Review. Trop. Anim. Health Prod..

[44] van Broekhoven S. (2008). Quality and Safety Aspects of Mealworms as Human Food.

[45] Toviho O.A. (2022). Nutrient Composition and Growth of Yellow Mealworm (*Tenebrio molitor*) at Different Ages and Stages of the Life Cycle. Agriculture.

[46] Hosen M., Khan A.R., Hossain M. (2004). Growth and Development of the Lesser Mealworm, *Alphitobius Diaperinus* (Panzer) (Coleoptera: Tenebrionidae) on Cereal Flours. Pak. J. Biol. Sci..

[47] Rumbos C.I., Bliamplias D., Gourgouta M., Michail V., Athanassiou C.G. (2021). Rearing *Tenebrio molitor* and *Alphitobius diaperinus* larvae on seed cleaning process byproducts. Insects.

[48] Rumbos C.I., Karapanagiotidis I.T., Mente E., Athanassiou C.G. (2019). The Lesser Mealworm *Alphitobius Diaperinus*: A Noxious Pest or a Promising Nutrient Source?. Rev. Aquac..

[49] Mariod A.A., Mirghani M.E.S., Hussein I., Mariod A.A., Mirghani M.E.S., Hussein I. (2017). Tenebrio molitor Mealworm. Unconventional Oilseeds and Oil Sources.

[50] Gomaa K.F.S. (2017). Evaluation of Five Pearl Millet Ecotypes Susceptibility to the Nymphal Instars of Migratory Locust. Trop. Drylands.

[51] Rumbos C.I., Karapanagiotidis I.T., Mente E., Psofakis P., Athanassiou C.G. (2020). Evaluation of Various Commodities for the Development of the Yellow Mealworm, *Tenebrio molitor*. Sci. Rep..

[52] Dreyer M., Hörtenhuber S., Zollitsch W., Jäger H., Schaden L.M., Gronauer A., Kral I. (2021). Environmental Life Cycle Assessment of Yellow Mealworm (*Tenebrio molitor*) Production for Human Consumption in Austria—A Comparison of Mealworm and Broiler as Protein Source. Int. J. Life Cycle Assess..

[53] Zielińska E., Baraniak B., Karaś M., Rybczyńska K., Jakubczyk A. (2015). Selected Species of Edible Insects as a Source of Nutrient Composition. Food Res. Int..

[54] Jantzen da Silva Lucas A., Menegon de Oliveira L., da Rocha M., Prentice C. (2020). Edible Insects: An Alternative of Nutritional, Functional and Bioactive Compounds. Food Chem..

[55] Hong J., Han T., Kim Y.Y. (2020). Mealworm (*Tenebrio molitor* Larvae) as an Alternative Protein Source for Monogastric Animal: A Review. Animals.

[56] Benzertiha A., Kierończyk B., Kołodziejski P., Pruszyńska–Oszmałek E., Rawski M., Józefiak D., Józefiak A. (2020). *Tenebrio molitor* and *Zophobas Morio* Full-Fat Meals as Functional Feed Additives Affect Broiler Chickens’ Growth Performance and Immune System Traits. Poult. Sci..

[57] Ravzanaadii N., Kim S.-H., Choi W.-H., Hong S.-J., Kim N.-J. (2012). Nutritional Value of Mealworm, *Tenebrio molitor* as Food Source. Int. J. Ind. Entomol..

[58] Heidari-Parsa S., Imani S., Fathipour Y., Kheiri F., Chamani M. (2018). Determination of Yellow Mealworm (*Tenebrio molitor*) Nutritional Value as an Animal and Human Food Supplementation. Arthropods.

[59] Boulos S., Tännler A., Nyström L. (2020). Nitrogen-to-Protein Conversion Factors for Edible Insects on the Swiss Market: *T. molitor*, *A. domesticus*, and *L. migratoria*. Front. Nutr..

[60] Kotsou K., Chatzimitakos T., Athanasiadis V., Bozinou E., Rumbos C.I., Athanassiou C.G., Lalas S.I. (2023). Enhancing the Nutritional Profile of *Tenebrio molitor* Using the Leaves of *Moringa oleifera*. Foods.

[61] Gkinali A., Matsakidou A., Paraskevopoulou A. (2022). Characterization of *Tenebrio molitor* Larvae Protein Preparations Obtained by Different Extraction Approaches. Foods.

[62] Siulapwa N., Mwambungu A., Lungu E., Sichilima W. (2012). Nutritional Value of Four Common Edible Insects in Zambia. Int. J. Sci. Res..

[63] Aberoumand A., Baesi F. (2023). The Nutritional Quality and Contents of Heavy Elements Due to Thermal Processing and Storage in Canned *Thunnus tonggol* Fish Change Compared to Fresh Fish. Food Sci. Nutr..

[64] Jensen I.J., Bodin N., Govinden R., Elvevoll E.O. (2023). Marine Capture Fisheries from Western Indian Ocean: An Excellent Source of Proteins and Essential Amino Acids. Foods.

[65] Adámková A., Mlček J., Adámek M., Borkovcová M., Bednářová M., Hlobilová V., Knížková I., Juríková T. (2020). *Tenebrio molitor* (Coleoptera: Tenebrionidae)—Optimization of Rearing Conditions to Obtain Desired Nutritional Values. J. Insect Sci..

[66] Machona O., Matongorere M., Chidzwondo F., Mangoyi R. (2022). Evaluation of Nutritional Content of the Larvae of *Tenebrio molitor*, and Formulation of Broiler Stockfeed. Entomol. Appl. Sci. Lett..

[67] Wu R.A., Ding Q., Yin L., Chi X., Sun N., He R., Luo L., Ma H., Li Z. (2020). Comparison of the Nutritional Value of Mysore Thorn Borer (*Anoplophora chinensis*) and Mealworm Larva (*Tenebrio molitor*): Amino Acid, Fatty Acid, and Element Profiles. Food Chem..

[68] Ao X., Yoo J.S., Wu Z.L., Kim I.H. (2020). Can Dried Mealworm (*Tenebrio molitor*) Larvae Replace Fish Meal in Weaned Pigs?. Livest. Sci..

[69] Yoo J.S., Cho K.H., Hong J.S., Jang H.S., Chung Y.H. (2018). Nutrient Ileal Digestibility Evaluation of Dried *Tenebrio molitor*. Asian Australas. J. Anim. Sci..

[70] Dreassi E., Cito A., Zanfini A., Materozzi L., Botta M., Francardi V. (2017). Dietary Fatty Acids Influence the Growth and Fatty Acid Composition of the Yellow Mealworm *Tenebrio molitor* (Coleoptera: Tenebrionidae). Lipids.

[71] Di Pasquale M.G. (2009). The Essentials of Essential Fatty Acids. J. Diet. Suppl..

[72] Lawal K.G., Kavle R.R., Akanbi T.O., Mirosa M., Agyei D. (2021). Enrichment in Specific Fatty Acids Profile of *Tenebrio molitor* and *Hermetia illucens* Larvae through Feeding. Futur. Foods.

[73] Ali M.Y., Sina A.A.I., Khandker S.S., Neesa L., Tanvir E.M., Kabir A., Khalil M.I., Gan S.H. (2021). Nutritional Composition and Bioactive Compounds in Tomatoes and Their Impact on Human Health and Disease: A Review. Foods.

[74] Ghosh S., Lee S.M., Jung C., Meyer-Rochow V.B. (2017). Nutritional Composition of Five Commercial Edible Insects in South Korea. J. Asia Pac. Entomol..

[75] Meyer-Rochow V.B. (1975). Can Insects Help to Ease the Problem of World Food Shortage?. Search.

[76] Errico S., Spagnoletta A., Verardi A., Moliterni S., Dimatteo S., Sangiorgio P. (2022). *Tenebrio molitor* as a Source of Interesting Natural Compounds, Their Recovery Processes, Biological Effects, and Safety Aspects. Compr. Rev. Food Sci. Food Saf..

[77] Sete da Cruz R.M., da Silva C., da Silva E.A., Hegel P., Barão C.E., Cardozo-Filho L. (2022). Composition and Oxidative Stability of Oils Extracted from *Zophobas morio* and *Tenebrio molitor* Using Pressurized N-Propane. J. Supercrit. Fluids.

[78] Alves A.V., Freitas de Lima F., Granzotti da Silva T., de Oliveira V.S., Kassuya C.A.L., Sanjinez-Argandoña E.J. (2019). Safety Evaluation of the Oils Extracted from Edible Insects (*Tenebrio molitor* and *Pachymerus Nucleorum*) as Novel Food for Humans. Regul. Toxicol. Pharmacol..

[79] Gantner M., Kr K., Piotrowska A., Sionek B., Sadowska A. (2022). Adding Mealworm (*Tenebrio molitor* L.) Powder to Wheat Bread: Effects on Physicochemical, Sensory and Microbiological Qualities of the End-Product. Molecules.

[80] Wu R.A., Ding Q., Lu H., Tan H., Sun N., Wang K., He R., Luo L., Ma H., Li Z. (2020). Caspase 3-Mediated Cytotoxicity of Mealworm Larvae (*Tenebrio molitor*) Oil Extract against Human Hepatocellular Carcinoma and Colorectal Adenocarcinoma. J. Ethnopharmacol..

[81] Purschke B., Stegmann T., Schreiner M., Jäger H. (2017). Pilot-scale Supercritical CO_2_ Extraction of Edible Insect Oil from *Tenebrio molitor* L. Larvae—Influence of Extraction Conditions on Kinetics, Defatting Performance and Compositional Properties. Eur. J. Lipid Sci. Technol..

[82] Kim Y.M. (2017). Quality characteristics of white bread with *Tenebrio molitor* linne powder. Korean J. Food Nutr..

[83] Mancini S., Fratini F., Tuccinardi T., Degl’Innocenti C., Paci G. (2020). *Tenebrio molitor* Reared on Different Substrates: Is It Gluten Free?. Food Control.

[84] Harrison J.F. (2001). Insect Acid-Base Physiology. Annu. Rev. Entomol..

[85] Roncolini A., Milanović V., Cardinali F., Osimani A., Garofalo C., Sabbatini R., Clementi F., Pasquini M., Mozzon M., Foligni R. (2019). Protein Fortification with Mealworm (*Tenebrio molitor* L.) Powder: Effect on Textural, Microbiological, Nutritional and Sensory Features of Bread. PLoS ONE.

[86] Commission Regulation (EC) (2007). N0.1441/2007 Amending Regulation (EC) No 2073/2005 on Microbiological Criteria for Foodstuffs. Off. J. Eur. Union.

[87] Khuenpet K., Pakasap C., Vatthanakul S., Kitthawee S. (2020). Effect of Larval—Stage Mealworm (*Tenebrio molitor*) Powder on Qualities of Bread. Int. J. Agric. Technol..

[88] Ahmad S., Naz A., Usman M., Amjad A., Pasha I., Farooq U. (2022). Impediment Effect of Chemical Agents (Additives) on Gluten Development in Cookie Dough. J. Food Sci. Technol..

[89] Zielińska E., Pankiewicz U. (2020). Nutritional, Physiochemical, and Antioxidative Characteristics of Shortcake Biscuits Enriched with *Tenebrio molitor* Flour. Molecules.

[90] Xie X., Yuan Z., Fu K., An J., Deng L. (2022). Effect of Partial Substitution of Flour with Mealworm (*Tenebrio molitor* L.) Powder on Dough and Biscuit Properties. Foods.

[91] Mihaly Cozmuta A., Uivarasan A., Peter A., Nicula C., Kovacs D.E., Mihaly Cozmuta L. (2023). Yellow Mealworm (*Tenebrio molitor*) Powder Promotes a High Bioaccessible Protein Fraction and Low Glycaemic Index in Biscuits. Nutrients.

[92] Biltoft-Jensen A., Matthiessen J., Ygil K.H., Christensen T. (2022). Defining Energy-Dense, Nutrient-Poor Food and Drinks and Estimating the Amount of Discretionary Energy. Nutrients.

[93] Djouadi A., Sales J.R., Carvalho M.O., Raymundo A. (2022). Development of Healthy Protein-Rich Crackers Using *Tenebrio molitor* Flour. Foods.

[94] Tsao R. (2010). Chemistry and Biochemistry of Dietary Polyphenols. Nutrients.

[95] Bartkowicz J., Babicz-Zielińska E. (2020). Acceptance of Bars with Edible Insects by a Selected Group of Students from Tri-City, Poland. Czech J. Food Sci..

[96] Caparros Megido R., Gierts C., Blecker C., Brostaux Y., Haubruge É., Alabi T., Francis F. (2016). Consumer Acceptance of Insect-Based Alternative Meat Products in Western Countries. Food Qual. Prefer..

[97] Kim H.W., Setyabrata D., Lee Y.J., Jones O.G., Kim Y.H.B. (2016). Pre-Treated Mealworm Larvae and Silkworm Pupae as a Novel Protein Ingredient in Emulsion Sausages. Innov. Food Sci. Emerg. Technol..

[98] Hirschman C. (2013). The Contributions of Immigrants to American Culture. Daedalus.

[99] Choi Y.S., Kim T.K., Choi H.D., Park J.D., Sung J.M., Jeon K.H., Paik H.D., Kim Y.B. (2017). Optimization of Replacing Pork Meat with Yellow Worm (*Tenebrio molitor* L.) for Frankfurters. Korean J. Food Sci. Anim. Resour..

[100] Grossi G., Goglio P., Vitali A., Williams A.G. (2019). Livestock and Climate Change: Impact of Livestock on Climate and Mitigation Strategies. Anim. Front..

[101] Woo K.-L., Lee S.-C., Jang D.-K. (2003). Quality Characteristics of Soy Sauces Containing Shiitake Mushroom (*Lentinus edodes*). Appl. Biol. Chem..

[102] Nelson G., Chandrashekar J., Hoon M.A., Feng L., Zhao G., Ryba N.J., Zuker C.S. (2002). An Amino-Acid Taste Receptor. Nature.

[103] Choi J.M., Lee C.B., Kim H.S. (2016). Quality Characteristics of Soy Sauces by Various Manufacturing Methods. Culin. Sci. Hosp. Res..

[104] Cho J.H., Zhao H.L., Kim J.S., Kim S.H., Chung C.H. (2018). Characteristics of Fermented Seasoning Sauces Using *Tenebrio molitor* Larvae. Innov. Food Sci. Emerg. Technol..

[105] Lee H., Kim Y. (2022). Development of a Low-Salt Soy Sauce with Enhanced Flavor and Functionality with Soy Residue and *Tenebrio molitor* Larvae Powder. J. Food Process. Preserv..

[106] Doig A.J. (2017). Frozen, but No Accident—Why the 20 Standard Amino Acids Were Selected. FEBS J..

[107] Yoo J., Hwang J.-S., Goo T.-W., Yun E.-Y. (2013). Comparative Analysis of Nutritional and Harmful Components in Korean and Chinese Mealworms (*Tenebrio molitor*). J. Korean Soc. Food Sci. Nutr..

[108] Alvarez V.B., Clark S., Costello M., Drake M., Bodyfelt F. (2009). Ice Cream and Related Products. The Sensory Evaluation of Dairy Products.

[109] Alvarez V. (2023). Ice Cream and Frozen Desserts. Ullmann’s Encyclopedia of Industrial Chemistry.

[110] Balaji P., Shalini N., Santhasheela M., Vidhyavathi A. (2021). Consumer Choice of Ice Creams: A Binary Logit Model of Analysis. Madras Agric. J..

[111] Hernández Toxqui A.G., Ramírez Ramírez J., Pino Moreno J.M., Talamantes Gómez J.M., Angeles Campos S.C., Ramírez Orejel J.C. (2021). Development of Nutraceutical Ice Creams Using Flour Yellow Worm Larvae (*Tenebrio molitor*), Chia (*Salvia Hispanica*), and Quinoa (*Chenopodium Quinoa*). Front. Vet. Sci..

[112] Bus A., Worsley A. (2003). Consumers’ Sensory and Nutritional Perceptions of Three Types of Milk. Public Health Nutr..

[113] Smith T., Campbell R., Drake M. (2016). Sensory Properties of Milk Protein Ingredients. Advanced Dairy Chemistry: Volume 1B: Proteins: Applied Aspects.

[114] Tello A., Aganovic K., Parniakov O., Carter A., Heinz V., Smetana S. (2021). Product Development and Environmental Impact of an Insect-Based Milk Alternative. Futur. Foods.

[115] Opio C. (2017). The Global Livestock Environmental Assessment Model.

[116] Garcia-Fontanals L., Llorente R., Valderrama J., Bravo S., Talens C. (2023). Hybrid Spreadable Cheese Analogues with Faba Bean and Mealworm (*Tenebrio molitor*) Flours: Optimisation Using Desirability-Based Mixture Design. Foods.

[117] Martineau-Côté D., Achouri A., Karboune S., L’Hocine L. (2022). Faba Bean: An Untapped Source of Quality Plant Proteins and Bioactives. Nutrients.

[118] Son Y.J., Choi S.Y., Hwang I.K., Nho C.W., Kim S.H. (2020). Could Defatted Mealworm (*Tenebrio molitor*) and Mealworm Oil Be Used as Food Ingredients?. Foods.

[119] Kamal-Eldin A. (2006). Effect of Fatty Acids and Tocopherols on the Oxidative Stability of Vegetable Oils. Eur. J. Lipid Sci. Technol..

[120] Kalompatsios D., Athanasiadis V., Chatzimitakos T., Palaiogiannis D., Lalas S.I., Makris D.P. (2023). Sustainable Exploitation of Waste Orange Peels: Enrichment of Commercial Seed Oils and the Effect on Their Oxidative Stability. Waste.

[121] Rizvi S., Raza S.T., Ahmed F., Ahmad A., Abbas S., Mahdi F. (2014). The Role of Vitamin E in Human Health and Some Diseases. Sultan Qaboos Univ. Med. J..

[122] Maraschiello C., Sárraga C., García Regueiro J.A. (1999). Glutathione Peroxidase Activity, TBARS, and α-Tocopherol in Meat from Chickens Fed Different Diets. J. Agric. Food Chem..

[123] Surai P.F., Sparks N.H.C. (2000). Tissue-Specific Fatty Acid and α-Tocopherol Profiles in Male Chickens Depending on Dietary Tuna Oil and Vitamin E Provision. Poult. Sci..

[124] Okogeri O., Tasioula-Margari M. (2002). Changes Occurring in Phenolic Compounds and α-Tocopherol of Virgin Olive Oil during Storage. J. Agric. Food Chem..

[125] Gasco L., Dabbou S., Gai F., Brugiapaglia A., Schiavone A., Birolo M., Xiccato G., Trocino A. (2019). Quality and Consumer Acceptance of Meat from Rabbits Fed Diets in Which Soybean Oil Is Replaced with Black Soldier Fly and Yellow Mealworm Fats. Animals.

